# Evolutionary dynamics of *DIRS-like* and *Ngaro-like* retrotransposons in *Xenopus laevis* and *Xenopus tropicalis* genomes

**DOI:** 10.1093/g3journal/jkab391

**Published:** 2021-11-18

**Authors:** Camilla Borges Gazolla, Adriana Ludwig, Joana de Moura Gama, Daniel Pacheco Bruschi

**Affiliations:** 1 Departamento de Genética, Laboratório de Citogenética Evolutiva e Conservação Animal (LabCECA), Universidade Federal do Paraná, Curitiba, PR 80060-000, Brazil; 2 Departamento de Genética, Programa de Pós-Graduação em Genética (PPG-GEN), Universidade Federal do Paraná (UFPR), Curitiba, PR 80060-000, Brazil; 3 Laboratório de Ciências e Tecnologias Aplicadas em Saúde (LaCTAS), Instituto Carlos Chagas—Fiocruz-PR, Curitiba, PR 81350-010, Brazil

**Keywords:** Anura, transposable elements, DIRS, retrotransposon

## Abstract

Anuran genomes have a large number and diversity of transposable elements, but are little explored, mainly in relation to their molecular structure and evolutionary dynamics. Here, we investigated the retrotransposons containing tyrosine recombinase (YR) (order DIRS) in the genome of *Xenopus tropicalis* and *Xenopus laevis*. These anurans show 2*n* = 20 and the 2*n* = 36 karyotypes, respectively. They diverged about 48 million years ago (mya) and *X. laevis* had an allotetraploid origin (around 17–18 mya). Our investigation is based on the analysis of the molecular structure and the phylogenetic relationships of 95 DIRS families of *Xenopus* belonging to *DIRS-like* and *Ngaro-like* superfamilies. We were able to identify molecular signatures in the 5' and 3' noncoding terminal regions, preserved open reading frames, and conserved domains that are specific to distinguish each superfamily. We recognize two ancient amplification waves of *DIRS*-*like* elements that occurred in the ancestor of both species and a higher density of the old/degenerate copies detected in both subgenomes of *X. laevis*. More recent amplification waves are seen in *X. tropicalis* (less than 3.2 mya) and *X. laevis* (around 10 mya) corroborating with transcriptional activity evidence. All *DIRS-like* families were found in both *X. laevis* subgenomes, while a few were most represented in the L subgenome. *Ngaro-like* elements presented less diversity and quantity in *X. tropicalis* and *X. laevis* genomes, although potentially active copies were found in both species and this is consistent with a recent amplification wave seen in the evolutionary landscape. Our findings highlight a differential diversity-level and evolutionary dynamics of the YR retrotransposons in *X. tropicalis* and *X. laevis* species expanding our comprehension of the behavior of these elements in both genomes during the diversification process.

## Introduction

Transposable elements (TEs) are the most variable feature of the vertebrate genome, and their role in shaping genomic diversity has attracted considerable interest in recent years (Bourque *et al.* 2018; Wicker *et al.* 2018). An exceptional diversity of TEs has been reported in all the amphibian genomes sequenced so far (Hellsten *et al.* 2010; Sun *et al*. 2015; Jiang *et al.* 2015; Session *et al.* 2016; Hammond *et al.* 2017; Edwards *et al.* 2018; Seidl *et al.* 2019). In *Xenopus tropicalis*, a model organism for genomic studies, TEs represent approximately one-third of the genome (Hellsten *et al.* 2010). Despite the considerable abundance of TEs in genome annotation, the diversity, molecular structure, and evolutionary dynamics of these elements are still poorly understood. The DIRS elements are a good example of this richness that has not been explored.

Retrotransposons of the order DIRS are widely distributed in eukaryote genomes (Wicker *et al.* 2007), except for the birds and mammals (Poulter and Butler 2015). The unifying feature of these elements is that they encode a tyrosine recombinase (YR), which participates in the process of integrating the element into the genome (Poulter and Butler 2015). Other retrotransposons employ endonucleases (LINEs and PLEs) or DDE-type integrase [long terminal repeats (LTRs)] (Wicker *et al.* 2007).

The DIRS elements were named in recognition of the first retrotransposon containing YR to be described, *DIRS-1*, which was found in the slime mold *Dictyostelium discoideum* (Cappello *et al.* 1985). This order can be divided into four superfamilies based on sequence structure and phylogeny: *DIRS-like, Ngaro-like, PAT-like*, and *VIPER-like* (Ribeiro *et al.* 2019). In general, the DIRS elements have three open reading frames (ORFs). The first ORF corresponds to a gag-like domain, the second corresponds to the reverse transcriptase (RT) and RNAse H (RH), and the third corresponds to the YR. Another characteristic of these elements is that the ORFs frequently overlap and have terminal repeats that vary in structure among the superfamilies (Poulter and Goodwin 2005; Ribeiro *et al.* 2019).

The *DIRS-like* elements can present a conserved methyltransferase (MT) domain downstream from the RT/RH, although the function of this domain is still unknown (Goodwin *et al.* 2004; Poulter and Butler 2015). The noncoding portion varies in its sequence among the elements, although its basic structure is composed of inverted terminal repeats (ITRs) and an internal complementary region (ICR), which is complementary to the beginning of ITR5′ and the end of the ITR3′ (Cappello *et al.* 1985; Poulter and Butler 2015).

The *Ngaro-like* elements were described after the *DIRS-like* and are distinguished by their split direct repeats (SDR), composed of A1 in the 5′ end and B1, A2, and B2 in the 3′ end, where A1 and A2 are identical, as are B1 and B2 (Goodwin *et al.* 2004). These elements do not contain the MT-like domain found in the *DIRS-like*, although in amphibians, they contain an ORF encoding a hydrolase domain (Hydro–SGNH) after the YR, but with no proven function (Goodwin and Poulter 2004; Poulter and Butler 2015).

The *PAT-like* elements are phylogenetically closely related to the *DIRS-like* elements (Goodwin and Poulter 2001; Goodwin and Poulter 2004; Poulter and Goodwin 2005), although these two groups are not always monophyletic (Ribeiro *et al.* 2019), and they can be differentiated by structural variations in the terminal repeats. Such as for *Ngaro-like*, *PAT-like* elements are composed of SDRs (Poulter and Goodwin 2005; Ribeiro *et al.* 2019). The *VIPER-like* elements also have SDRs, and form a distinct group of retrotransposons restricted to the protozoans of the order Kinetoplastida (Ribeiro *et al.* 2019).

In the Anura, both *DIRS-like* and *Ngaro-like* elements have been described in *X. tropicalis* and *Xenopus* *laevis* (Goodwin *et al.* 2004; Hellsten *et al.* 2010; Poulter and Butler 2015). These species are found across sub-Saharan Africa and have an aquatic life that distinguishes them from other anurans (Hellsten *et al.* 2010). The *X. tropicalis* karyotype is composed of 2*n* = 20 chromosomes with an estimated genome size of 1.7 Gbp (Hellsten *et al.* 2010), while the *X. laevis* karyotype has a diploid number of 2*n* = 36 chromosomes, which originated from a process of allopolyploidy, with an estimated size of 3.1 Gbp where the two subgenomes (called S and L) are identified (Session *et al.* 2016). The available estimates indicate that 1% of the *X. tropicalis* genome is composed of distinct families of DIRS, some of which may still be active (Hellsten *et al.* 2010). Evidence of the transcriptional activity of the DIRS elements has already been found in both species (Poulter and Butler 2015), which highlights the possible role of these elements in genome function and evolution.

In the present study, we evaluated the diversity, molecular structure, and evolutionary dynamics of the elements of the order DIRS in *X. tropicalis* and *X. laevis*. We identified the structural characteristics of the YR retrotransposons of the DIRS order in both genomes, and described diagnostic characteristics for the best differentiation of the elements of the *DIRS-like* and *Ngaro-like* and evaluated the evolutionary dynamics of these superfamilies in these genomes.

## Materials and methods

An extract containing all the elements identified as DIRS was obtained from the Rebpase database (Jurka 2000) version 23.11. All the sequences from *X. tropicalis* and *X. laevis* were selected and analyzed using the NCBI “Open Reading Frame Finder” (ORFfinder) (https://www.ncbi.nlm.nih.gov/orffinder/) to identify ORFs with default parameters [“minimal ORF length (nt)” = 75; “Genetic code”: 1. Standard; “ORF start codon to use”: ATG only]. The presence of conserved domains was analyzed using the NCBI “Conserved Domains Search Service” (CD-Search) (Marchler-Bauer and Bryant 2004) with an e-value threshold adjusted to 0.1. The presence of ITRs, ICRs, and SDRs was investigated using NCBI BLASTn with the same sequence as query and subject, selecting the options “Align two or more sequences” and “somewhat similar sequences (blastn)”, with the “word size” parameter being adjusted to the minimum available for each sequence and the e-value threshold was 10.

Three families of *X. tropicalis* were selected for the analysis of copies in the genome, including one *DIRS*-*like* family (DIRS-37_XT) and two *Ngaro*-*like* families (DIRS-53_XT and DIRS-54_XT). We chose the DIRS-37_XT and DIRS-53_XT families as queries because they have the conserved structure of the ORFs and the complete domains, as well as the characteristic repeats for each superfamily. The DIRS-54_XT family was also used as a query to expand the searches of *Ngaro-like* even despite not having conserved terminal repeats.

The amino acid (aa) sequences corresponding to the RTs of both elements were used as queries in online tBLASTn searches against the *X. tropicalis* (GCA_000004195.4) and *X. laevis* (GCA_017654675.1) genomes. The first 10 hits were retrieved with 3 kb of both upstream and downstream regions. All the copies retrieved were analyzed for the identification of the ORFs, the conserved domains, and the repetitive regions as described above.

The evolutionary analyses were based on the alignment of the RT aa sequences, including the following sequences: (1) the consensus sequences of the DIRS families of *X. laevis* and *X. tropicalis* recovered from Rebpase; (2) the copies that are homologous to the DIRS-37_XT, DIRS-53_XT, and DIRS-54_XT families retrieved from the *X. tropicalis* and *X. laevis* genomes; and (3) elements known to belong to the different superfamilies of the order DIRS, *Ngaro-like—*Ngaro1_DR (AY152729—*Danio rerio*) and Lv_Ngaro2 (AGCV01398517—*Lytechinus variegatus*), *PAT*-*like—*SkowPAT (Rebpase—*Saccoglossus kowalevskii*), and *PAT* (Q26106—*Panagrellus redivivus*), and *DIRS*-*like—*DIRS-1_Acar (Rebpase—*Anolis carolinensis*) and DIRS-5_CBP (Rebpase—*Chrysemys picta bellii*).

The sequences were aligned using the PSI-coffee tool (Notredame *et al.* 2000), with Genedoc 2.7 (Nicholas and Nicholas 1997) being used for sequence manipulation and editing. Most of the RT sequences contained around 120 aa, and sequences with less than 70% coverage were excluded from the matrix. The MegaX program (Kumar *et al.* 2018) was used to determine the best aa substitution model. A distance tree was constructed using the Neighbor-joining method with JTT + G model and bootstrap test with 1000 replicates. A phylogenetic tree was also reconstructed using Bayesian inference, run in MrBayes 3.2.6 (Ronquist *et al.* 2012) based on the LG + G model. The Markov Chain Monte Carlo was run for 10,000,000 generations, sampled every 1000 generations, with 25% of the initial results being discarded as burn-in. The final trees were visualized and edited using iTOL (Letunic and Bork 2019).

The copies were named *a priori* according to the family used as the query, abbreviated to D37, D53, and D54, followed by the number of the copy referring to the order in which the sequence was recovered, while “XT” and “XL” are acronyms for *X. tropicalis* and *X. laevis*, respectively.

For the evolutionary landscape analysis, the consensus sequences available in the Repbase for each *DIRS*-*like* family of *X. tropicalis* and *X. laevis* and each *Ngaro-like* family of *X. tropicalis* were used to compose the libraries of each species. In the case of the *Ngaro-like* elements of *X. laevis*, as the families were not available in the Rebpase, the consensus sequences were obtained from the copies recovered in the genomic search described above. For that, the sequences were aligned using MAFFT v7.471, with the pairwise divergence being assessed using Genedoc 2.7 (Nicholas and Nicholas 1997) separating the sequences with more than 80% divergence into distinct groups (considering as different families). The consensus sequence of each group was obtained using UGENE (simple extended algorithm) with a 50% threshold (Okonechnikov *et al.* 2012). The *DIRS-like* and *Ngaro-like* libraries of each species were used to screen the genomes using RepeatMasker 4.1.0 (with the “-s,” “-nolow,” “-no_is,” “-a,” and “-lib” options). For *X. laevis*, the subgenomes S and L were screened separately. RepeatMasker utility Perl scripts were used to summarize the output (script buildSummary.pl) and to calculate Kimura 2-Parameter (K) divergence with adjusted CpG (script calcDivergenceFromAlign.pl). The scatter plot graphs representing the repeat evolutionary landscape were created using the Python Matplotlib-v3.3.2 (Hunter 2007) and edited in Inkscape software.

The age of the copies was estimated based on the time since the divergence of the ancestral sequence (since the consensus of each family used in the RepeatMasker is an approximation of its ancestor) using the formula: T = K/r (Jiang *et al.* 2002), where a divergence (K) was obtained as described above, and r is the nucleotide substitution rate of 3.1 × 10^−9^ substitutions per year, which is the average of the estimated substitution rates for *X. tropicalis* and *X. laevis*, and for the L and S subgenera of *X. laevis* (Session *et al.* 2016).

In order to investigate which families are being expressed in *X. tropicalis* and *X. laevis*, the expressed sequence tags (EST) library (Bowes *et al*. 2010) from both species were retrieved from Xenbase (http://www.xenbase.org/, RRID: SCR_003280) and the different DIRS families of both species were used as queries in BLASTn. The results were filtered by identity (>85%) and size (>100 bp).

## Results

### Xenopus DIRS sequences belong to *DIRS-like* and *Ngaro-like* families

A total of 75 YR-retroelement families were identified in the Rebpase for *X. tropicalis* and 20 for *X. laevis* (Supplementary Table S1). In the Repbase classification, all YR-containing elements are classified as superfamily DIRS, a final group within the LTR retrotransposons group (Kapitonov and Jurka 2008). Mainly concerning the YR elements, the Wicker *et al.* (2007) classification is more detailed, separating them into a distinct group of retrotransposons (order DIRS) and discriminating the clear distinct subgroups of DIRS into three superfamilies and more recently separation into four superfamilies has been suggested (Ribeiro *et al.* 2019). We thus previously assume that these DIRS families available in the Repbase could belong to any of the DIRS superfamilies, then our analyses indicate they belong only to *DIRS-like* and *Ngaro-like*.

**Figure 1:**
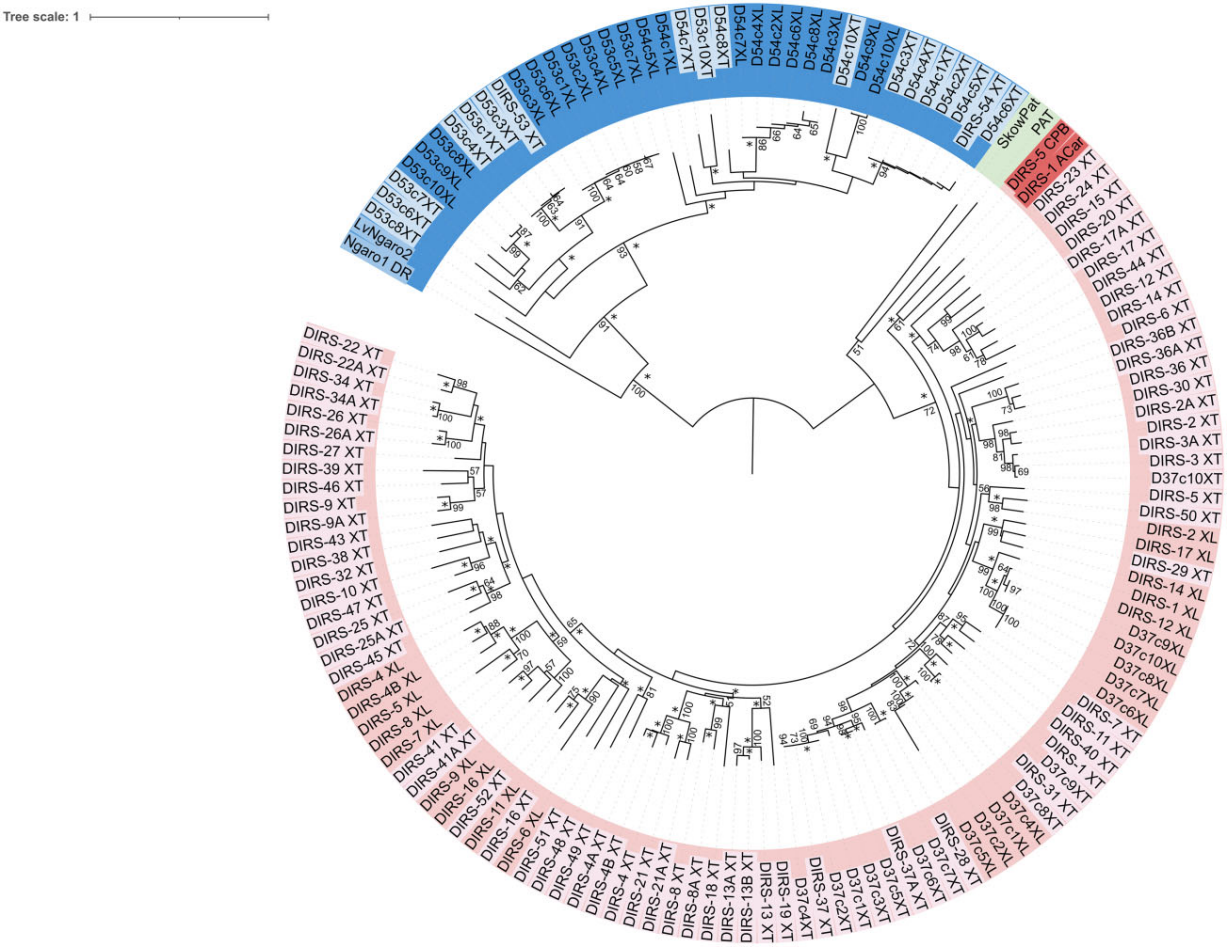
Sequence tree produced by neighbor-joining method (JTT + G), based on the amino acid sequences of the reverse transcriptase domain. The matrix was composed of the sequences of *X. tropicalis* and *X. laevis* DIRS elements obtained from the Rebpase database, the copies retrieved from both genomes and diagnostic sequences from each DIRS superfamily. The bootstrap values higher than 50 are indicated at the branches. The * sign near the nodes indicates the clade was supported with posterior probability higher than 80 in the Bayesian tree. Sequences of different superfamilies are highlighted in different colors and shades of each color also distinguish the sequences of *X. tropicalis* (XT) and *X. laevis* (XL).

The evolutionary trees based on the RT domain of all the elements present similar topologies and recovered two well-supported clades (Figure  1 and Supplementary Figure S1) in which all the *Xenopus* sequences grouped in either (1) a *DIRS*-*like* or (2) a *Ngaro-like* group. The two *PAT-like* sequences were not grouped as a monophyletic group.

The divergence between these two groups of sequences is clear (Figure  1). We recognized only two of the 95 *Xenopus* Rebpase DIRS families as belonging to the *Ngaro*-*like* superfamily, *i.e.*, DIRS-53_XT and DIRS-54_XT. Sequences from the families DIRS-6A_XT, DIRS-13C_XT, DIRS-27A_XT, DIRS-35_XT, DIRS-42_XT, DIRS-3_XL, DIRS-10_XL, DIRS-13_XL, DIRS-15_XL, DIRS-18_XL, and DIRS-19_XL were not included in the tree because the RT domain was too short, but all these elements present a *DIRS*-*like* terminal repeat pattern.

**Figure 2:**
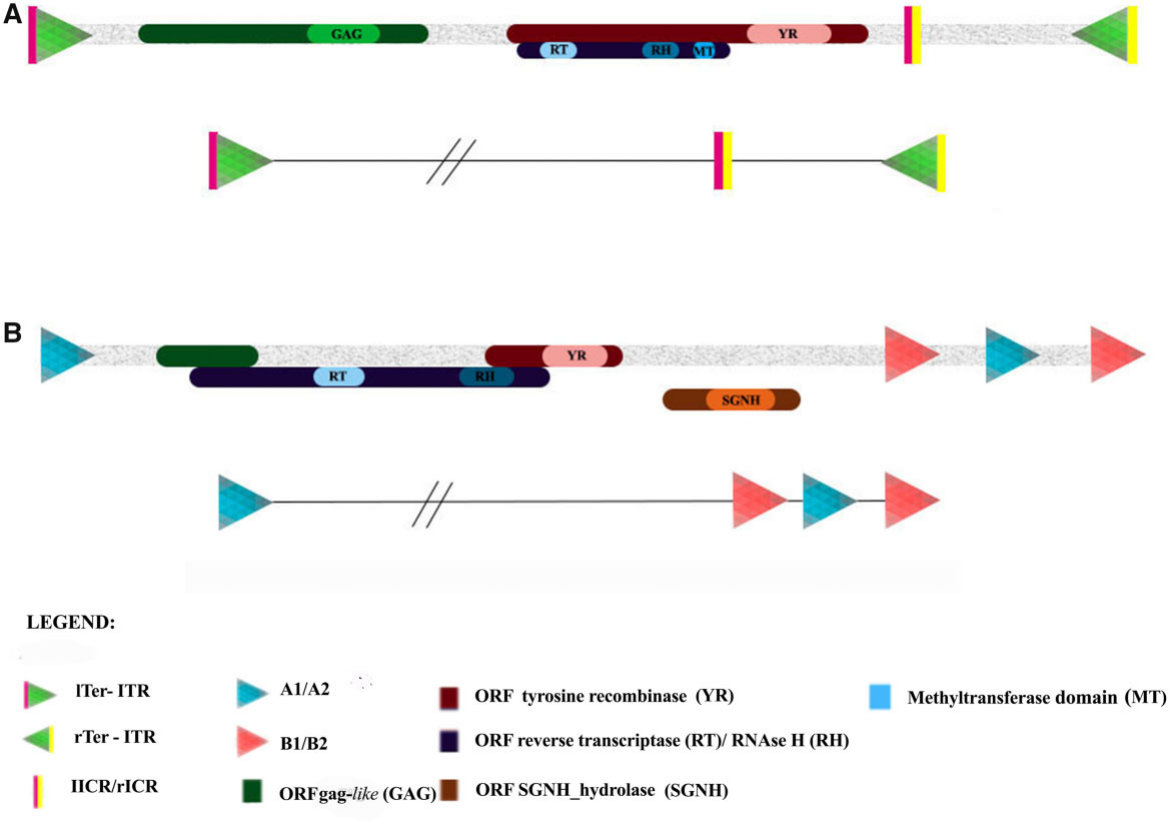
Schematic structure of the potentially complete *DIRS-like* and *Ngaro-like* retroelements of the *Xenopus tropicalis* genome. (A) Representation of the *X. tropicalis DIRS-like* elements, based on the Rebpase consensus sequences of the DIRS-37_XT, that contain three ORFs, conserved domains (gag, RT/RH/MT, and YR) and noncoding portions: inverted terminal repeats (ITRs) and internal complementary regions (ICRs). The expanded scheme of the terminal regions is shown in the lower plot. (B) Representation of the *X. tropicalis Ngaro-like* elements based on the Rebpase consensus sequences of the DIRS-53_XT that contain four ORFs, conserved domains (RT/RH, YR, and SGNH) and split direct repeats (SDRs).

The sequences of both superfamilies were analyzed, and a high level of congruence was found in the sequence structure in comparison with the DIRS families described in vertebrates (Goodwin and Poulter 2004) (Figure  2). These findings will be discussed below.

### DIRS-like families

The Repbase sequences of the *DIRS*-*like* superfamily found in the *X. tropicalis* and *X. laevis* genomes range from 4146 base pairs (bp) in DIRS-33_XT to 6224 bp in DIRS-2A_XT. We recognized three ORFs in almost all the families with several levels of overlap, involving primarily ORF2 and ORF3 (see Supplementary Tables S1 and S2, for more details).

The ORF1 encodes a gag-like protein and a LAP2alpha domain (∼650 aa) was predicted in all families. The ORF2 corresponds to the RT and RH domains, with around 120 and 356 aa, respectively. A deoxy-adenosine methylase (DAM/MT) domain of around 284 aa was also observed in the ORF2 of 38 of the families evaluated here. The ORF3 encodes the YR protein with a conserved DNA_BRE_C domain of around 584 aa (Supplementary Table S2).

The *DIRS*-*like* elements have 5′ and 3′ ITRs and an ICR region (Figure  2A), and this pattern of repeats was found in almost all the *Xenopus DIRS*-*like* families evaluated here. The ITRs have ∼120 bp and present a few nucleotide substitutions or indels between the left ITR (lITR) and the right ITR (rITR) (Figure  2A). The ICR is composed of two short sequences (lICR and rICR), which are complementary to the 5′ (lTer) and the 3′ (rTer) ends of the element (Figure  2A). The ICR and ITR sequences overlap slightly in most families.

Overall, 36 of the 75 Rebpase *DIRS*-*like* families of *X. tropicalis* present some level of degeneration in the molecular structure of the terminal repeats and/or ORFs domains (Supplementary Table S2). In *X. laevis*, 13 of the 20 families present premature interruptions in the ORFs or incomplete repeats (Supplementary Table S2), which indicates a high level of degeneration in these families. Concerning the EST data, we observed that most families present transcripts (55 families from *X. tropicalis* and 12 families from *X. laevis*) (Supplementary Table S1). The *DIRS*-*like* families of both genomes have characteristic thymine trinucleotides (*i.e.*, “TTT”) in both their 5′ and 3′ ends.

The RT sequence tree highlights the high level of family diversity of the *DIRS*-*like* clade in *Xenopus* (Figure  1). The diagnostic *DIRS-like* sequences from Sauropsids (DIRS-1_ACar and DIRS-5_CBP) were recovered as a basal branch, which indicates that most of *DIRS*-*like* families’ diversity was originated after the separation of amniotes and amphibians, although in the BA tree (Supplementary Figure S1), these sequences were located inside the *Xenopus DIRS-like* clade.

As the Rebpase nomenclature of the families established for a species follows the order of their description (Kapitonov and Jurka 2008; Bao *et al.* 2009), the evolutionary relationships among the families must be interpreted based on their phylogenetic relationships in the sequence trees, rather than their nomenclature in the databases. For example, DIRS-4_XT is not closely related to DIRS-4_XL, whereas DIRS-2_XL and DIRS-50_XT have a very close relationship. We recovered families of *DIRS*-*like* that were shared between the two species, such as DIRS-29_XT with DIRS-14_XL + DIRS-17_XL, DIRS-2_XL and DIRS-50_XT, DIRS-11_XL with DIRS-52_XT + DIRS-16_XT, and DIRS-41_XT + DIRS-41A_XT with DIRS-9_XL + DIRS-16_XL.

**Figure 3:**
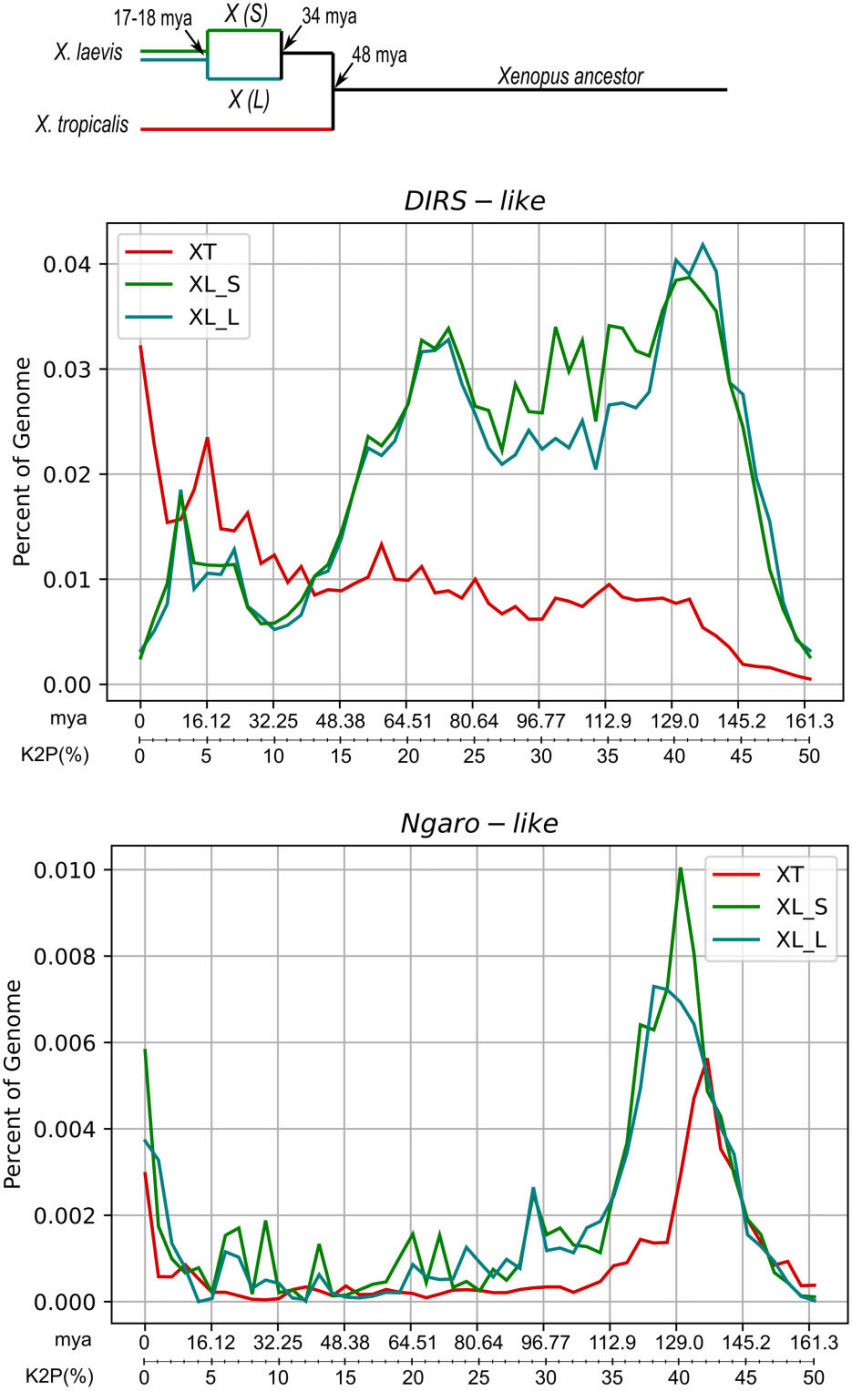
Evolution of *Xenopus* species and evolutionary dynamics of DIRS elements. The evolutionary events as estimated by Session *et al.* (2016) are shown: the speciation of *X. tropicalis* and the ancestor of *X. laevis* at 48 mya, the speciation of the L and S progenitors of *X. laevis* at 34 mya, and their hybridization around 17–18 mya. The graphs show the divergence of *DIRS*-*like* (above) and *Ngaro*-*like* (below) copies mapped in the genomes of *X. tropicalis* and *X. laevis* (S and L subgenomes) with their consensus sequence expressed in Kimura-2-parameters distance and the corresponding time of divergence in million years (*x*-axis) plotted in relation to the proportion in the genome (*y*-axis).

We also observed marked species-specific structuring in the sequence tree, recovering subclades that grouped families only from *X. tropicalis* or *X. laevis.* This indicates that many of the families may have originated after the separation of the two species, in particular in *X. tropicalis*. The evolutionary landscape profile observed in each genome further reinforces this conclusion (Figure  3). For *X. laevis*, in both subgenomes, it is possible to observe two broad ancient waves of amplification [60–80 million years ago (mya) and 110–135 mya], whereas, in the *X. tropicalis* genome, there is a peak of very recent amplification, which occurred less than 3.2 mya. The younger copies in *X. laevis* genome are found in smaller proportions than in *X. tropicalis* and a small peak of recent amplification is seen around 10 mya. Although the diversity of the *DIRS-like* families is much lower in *X. laevis*, they make up a larger proportion of the genome (1.03%) than in *X. tropicalis* (0.5%) with similar proportion in both subgenomes (0.45% for S subgenome and 0.58% for L subgenome). The families DIRS-2_XL, DIRS-3_XL, DIRS-10_XL, DIRS-12_XL, DIRS-13_XL, and DIRS-14_XL present a slightly higher proportion of mapping (around 60%) in the L genome (Supplementary Figure S2).

From potentially active families, we chose to analyze the DIRS-37_XT copies in the genomes. In *X. tropicalis*, we can observe that most copies recovered (except copies 4 and 6) are putative functional presenting all ORFs and conserved ITRs and ICR. Copies 1–7 are the most closely related and grouped in the clade containing DIRS-37_XT, DIRS-37A_XT, and DIRS-28_XT, but only the copies 1, 2, and 4 grouped with the query. The search has also recovered copies that grouped with more distant families, DIRS-31_XT (copies 8 and 9) and DIRS-3_XT (copy 10).

In *X. laevis*, the closest described families to DIRS-37_XT are DIRS-14_XL, DIRS-17_XL, and DIRS-2_XL. The nearest sequences to the query are the copies 1, 2, 4, and 5 (copy 3 was not included in the tree) forming a clade with no known family, suggesting that these sequences may be copies of a *X. laevis* family that has yet to be established. Copies 6–10 grouped with DIRS-12_XL, which is closely related to DIRS-1_XL. We found conserved copies in both subgenomes.

### *Ngaro-like* families

The *Ngaro*-*like* families DIRS-53_XT and DIRS-54_XT also present the three expected ORFs (encoding *gag*-like elements, RT/RH, and YR) with an additional ORF encoding a protein containing the SGNH_hydrolase domain. The reading frames of all the ORFs overlap (Figure  2B; Supplementary Table S2). The *Ngaro*-*like* elements are known to have a different type of terminal repeat, the SDRs (Goodwin and Poulter 2004). This pattern can be observed in the DIRS-53_XT copy (Figure  2B). This element has A1 and A2 repeats of 236 bp and B1 and B2 repeats of 152 bp, both being 100% identical. No SDRs were found in the DIRS-54_XT. Although none of the *X. laevis* Rebpase DIRS families grouped with *Ngaro-like* clade, our searches in the genome revealed the presence of sequences homologous to DIRS-53_XT and DIRS-54_XT. *Ngaro-like* transcripts were found for both species (Supplementary Table S1).

The copies of DIRS-53_XT and DIRS-54_XT recovered from *X. tropicalis* genome varied considerably in the degree of conservation of their sequences and structure (Supplementary Table S2), ranging from well-conserved copies to highly degenerate ones, due primarily to the loss of all or part of their 5′ and/or 3′ SDRs. Copies 1, 2, 4, 8, and 9 of DIRS-53_XT and copies 5 and 10 of DIRS-54_XT are potentially active.

In *X. laevis*, we observed that 13 homologous copies of DIRS-53_XT/DIRS-54_XT are preserved, while some include a complete or partial loss of the 5' and/or 3' SDRs or broken ORF (Supplementary Table S2). Copies 2, 3, 4, 7, 8, and 9 recovered with DIRS-53_XL and copies 1, 2, 3, 4, 5, 8 and 10 recovered with DIRS-54_XL are potentially active.

The copies recovered from both species were included in the sequence tree (Figure  1; except those with a short RT domain—D53c2XT, D53c5XT, D53c9XT). Clearly, DIRS-53_XT and DIRS-54_XT are very divergent (∼65% over only 600 bp of good alignment) and form two major groups of *Ngaro-like* sequences; however, some of the relationships were not supported by the bootstrap values and the Bayesian tree (Supplementary Figure S1) has shown a distinct topology, grouping some of the copies recovered with DIRS-54_XT in both species as a basal clade together with DIRS-53_XT. In both trees, the structuring of subclades with species-specific groups suggests the activity of these families after the separation of *X. tropicalis and X. laevis*. While the SDRs are not present in the consensus canonical copy of DIRS-54_XT, they can be observed in copies 5 and 10, which indicates that this family is also potentially active in *X. tropicalis*.

Similar to what we observed for *DIRS-like*, *Ngaro*-*like* elements density in *X. laevis* (0.091%) is approximately double of the density seen for *X. tropicalis* (0.044%) and these proportions are 10 times smaller than observed for *DIRS-like* in both genomes. The *Ngaro*-*like* evolutionary landscape profile (Figure  3) shows a recent amplification signal (less than 3.2 mya) in *X. tropicalis* genome and *X. laevis* subgenomes. Additionally, in both species, it is possible to observe a very ancient amplification wave, dating from 120 to 145 mya, that is more prominent in *X. laevis* graph.

## Discussion

In this work, we presented a detailed analysis of the YR-containing elements (order DIRS) available in the Repbase23.11 for *X. tropicalis* and *X. laevis* classifying them as either into the *DIRS-like* or *Ngaro-like* superfamilies. We have provided a detailed description of the structural characteristics of these elements and found the specific molecular signature of each superfamily. *DIRS*-*like* and *Ngaro*-*like* elements present distinct diagnostic features in their 5′ and 3′ noncoding terminal regions with the same structural elements already recorded in other metazoans, fungi, and protists (Goodwin and Poulter 2001; Goodwin and Poulter 2004; Poulter and Goodwin 2005; Poulter and Goodwin 2015). Our detailed analysis of the elements corroborates what has been described for these superfamilies concerning the ORFs and domains (Goodwin and Poulter 2001; Poulter and Goodwin 2005). We found the three main ORFs for both superfamilies and a MT domain or a hydrolase domain was found in the *DIRS-like* and *Ngaro-like* families, respectively.

We also identified conserved thymine trinucleotides (“TTT”) at the 5′ and 3′ ends in the complete copies of the *DIRS-like* elements of the genomes. This is probably a general pattern of the *DIRS*-*like* elements found in terminal regions, which may be essential for transposing these elements (Malicki *et al.* 2020). A similar terminal signature has also been described for the DrDIRS1 element of *Tribolium castaneum*, which has either the trinucleotides “GTT” or dinucleotides “AA” (Goodwin *et al.* 2004). Given this, the recognition of the similarities in the molecular structure of these elements from the two species would also contribute to the assessment of the diversity and evolutionary history of these YR retrotransposons in other anuran genomes.

Based on the phylogenetic criteria and molecular structure, we found that both superfamilies are present in both genomes. The presence of *DIRS-like* and *Ngaro-like* was already reported for *X. tropicalis* (Hellsten *et al.* 2010). On the other hand, for *X. laevis* (Goodwin and Poulter 2004), the information was not clear and no *Ngaro*-*like* family was deposited in the Repbase dataset version that we analyzed. We found a much greater diversity and proportion of elements of the *DIRS-like* superfamily, in comparison with the *Ngaro-like* elements, in both genomes. This richness of *DIRS-like* elements in *Xenopus* is also higher than that found for the fish *D.* *rerio* (12 families) or the reptile *A.* *carolinensis*, which has 42 families (Piednoël *et al.* 2011). *DIRS*-*like* and *Ngaro-like* elements are widely distributed in a number of metazoan groups, including fish, amphibians, reptiles, and some fungus (Ruiz-Perez *et al.* 1996) and protists (Goodwin and Poulter 2001; Poulter and Goodwin 2005). Although there is no clear report of DIRS elements in some groups of species, we believe that the distribution of these retrotransposons is underestimated since they are frequently classified in the amount of the LTR retrotransposons group in the annotation of repetitive sequences based on the RepeatMasker tool.

Several sequences of both superfamilies are structurally complete in *X. tropicalis* and *X. laevis*. Thus, it is relevant to compare the evolutionary landscape pattern of these superfamilies in both genomes since these species have undergone distinct evolutionary processes after the split. The speciation of *X. tropicalis* and the *X. laevis* ancestor at around 48 mya (Session *et al.* 2016; Figure  3). The *X. laevis* had an allotetraploid origin (around 17–18 mya) from two extant diploid progenitors separated at around 34 mya, and currently has two homoeologous subgenomes (L and S). The L and S subgenomes have undergone profound intragenomic diversification, which is compatible with the absence of recombination between the homeologous chromosome pairs of each subgenome since the allotetraploidization event (Session *et al.* 2016). If we consider the divergence time and evolutionary rates estimated by Session *et al.* (2016) as the most realistic scenario of the evolutionary history of *Xenopus* genus, our time-scale estimate of DIRS evolution shows different patterns in *X. tropicalis* and *X. laevis* genomes.

For *DIRS-like*, looking at the *X. laevis* subgenomes graphs, it is possible to suggest that two ancient waves of amplification (60–80 mya and 110–135 mya) have occurred indicating the long-time of persistence of these elements. Considering the time of the species splitting, these waves probably occurred in the ancestor of both species, while these were less clear in *X. tropicalis* genome, suggesting a higher turnover of degenerate copies in *X. tropicalis* than in *X. laevis* after the separation of these species. All families were mapped in both *X. laevis* subgenomes and for a few families, the genomic density was slightly higher in the subgenome L. It is suggested that the S subgenome has undergone intrachromosomal rearrangements and extensive small-scale deletions that resulted in the reduction of the length of the S chromosomes in comparison with their L homeologs (Session *et al.* 2016). The small signature of a recent wave of amplification in both subgenomes occurred after the allotetraploid origin of *X. laevis* resulting in a small proportion of younger copies that have been maintenance preserved for a long-time in *X. laevis* genome. In *X. tropicalis*, the *DIRS-like* amplification waves were more prominent occurring around 16 mya and less than 3.2 mya. The existence of potentially active copies of *DIRS*-*like* identified here is in agreement with the evidence of transcriptional activity detected in the transcriptome data on *X. tropicalis* (Poulter and Butler 2015) and ours searches in the EST libraries of both species (Supplementary Table S1). Recent burst events in both species may also have contributed to the diversification of families in each genome, particularly in *X. tropicalis*, which is consistent with the strong species-specific grouping seen in the sequence tree.

The evolutionary landscape pattern and the phylogenetic trees highlight the relative success of the *DIRS*-*like* elements in the *Xenopus* genomes in comparison with the *Ngaro*-*like* superfamily that presents very low diversity and quantity. *Ngaro-like* had a very ancient amplification wave followed by a long period of senescence. Despite *Ngaro-like* elements have failed to increase copy number and diversify in *Xenopus*, some potentially active copies are found in both genomes what is consistent with the very recent amplification wave (less than 3.2 mya) seen in both species. The recent amplification indicates that *Ngaro-like* copies were maintained as active and somehow silenced for a long period of *Xenopus* evolution or were recently reactivated or reintroduced in these genomes.

It is not clear, however, the reason why the *Ngaro-like* did not achieve the same success as *DIRS-like*, but it could be related to the known differences in the transposition mechanism (Poulter and Goodwin 2005) of both superfamilies or to a possible variation in silencing efficiency by the host, while it could also be explained by chance. Still, evidence of the accumulation of DIRS-1 element on centromeres of the *D. discoideum* genome (Dubin *et al.* 2010; Malicki *et al.* 2017) could indicate their role during the centromeric heterochromatin biogenesis in this genome and open new perspectives to future evaluation about their biological significance in their success in *Xenopus* genomes.

The presence of ancient and senescent DIRS copies in both genomes is consistent with “TE cycle life” of the genome (Kidwell and Lisch 2001), in which ancient mobile elements may lose their autonomy and no amplifications occur, with the nucleotide sequences losing their identity, with the senescent elements eventually being deleted or becoming completely divergent. The intragenomic behavior of the TEs depends on the balance between repression and expression, due to the need to avoid a large number of copies becoming a disadvantage for the genome (Bourque *et al.* 2018). The conservation of the molecular structure of these elements is related directly to these genetic mechanisms, which determine either an increase or loss of TE diversity, depending on the repertoire of TEs, during the genomic evolution of each lineage.

So far, this is the most comprehensive work of DIRS retrotransposons in Amphibia being a starting point to guide research on the evolution and functionalities of these retrotransposons in other anuran genomes and in this way solve gaps in evolutionary history not only in the way they behaved during evolution but also in how they influenced their genomes.

## Data availability

Supplementary files available at Table S1 contain description of basic features of the DIRS families of *Xenopus tropicalis* (XT) and *X. laevis* (XL) deposited in Rebpase. The consensus sequence of each family was evaluated for the presence/absence of complete conserved ORFs containing the expected domains and the presence of complete structure of repeats (ITRs and ICR for *DIRS-like* and SDRs for *Ngaro-like*). The presence of ESTs is also shown for each family. Supplementary Table S2 contains summary analyzes of the copies retrieved of the *X.* *tropicalis* and *X. laevis* genomes. Supplementary Figure S1 contains sequence tree produced by Bayesian inference, based on the amino acid sequences of the RT domain. The matrix was composed of the sequences of *X. tropicalis* and *X. laevis* DIRS elements obtained from the Rebpase database, the copies retrieved from both genomes and diagnostic sequences from each DIRS superfamily and Supplementary Figure S2 contains the proportion of all *X. laevis DIRS-like* families in the subgenomes S and L. The authors affirm that all data necessary for confirming the conclusions of the article are present within the article, figures, and tables. Supplementary material available at figshare: https://doi.org/10.25387/g3.15105336.
